# Supplementary material for: Assessment of Human SARS CoV-2-Specific T-Cell Responses Elicited In Vitro by New Computationally Designed mRNA Immunogens (COVARNA)
Source: Vaccines (Basel). 2023 Dec 22;12(1):15. doi: 10.3390/vaccines12010015 (PMC10820377; doi:10.3390/vaccines12010015)
Supplement: Supplementary file 1 [file vaccines-12-00015-s001.zip › vaccines-2737485-supplementary.pptx]

## Slide 1
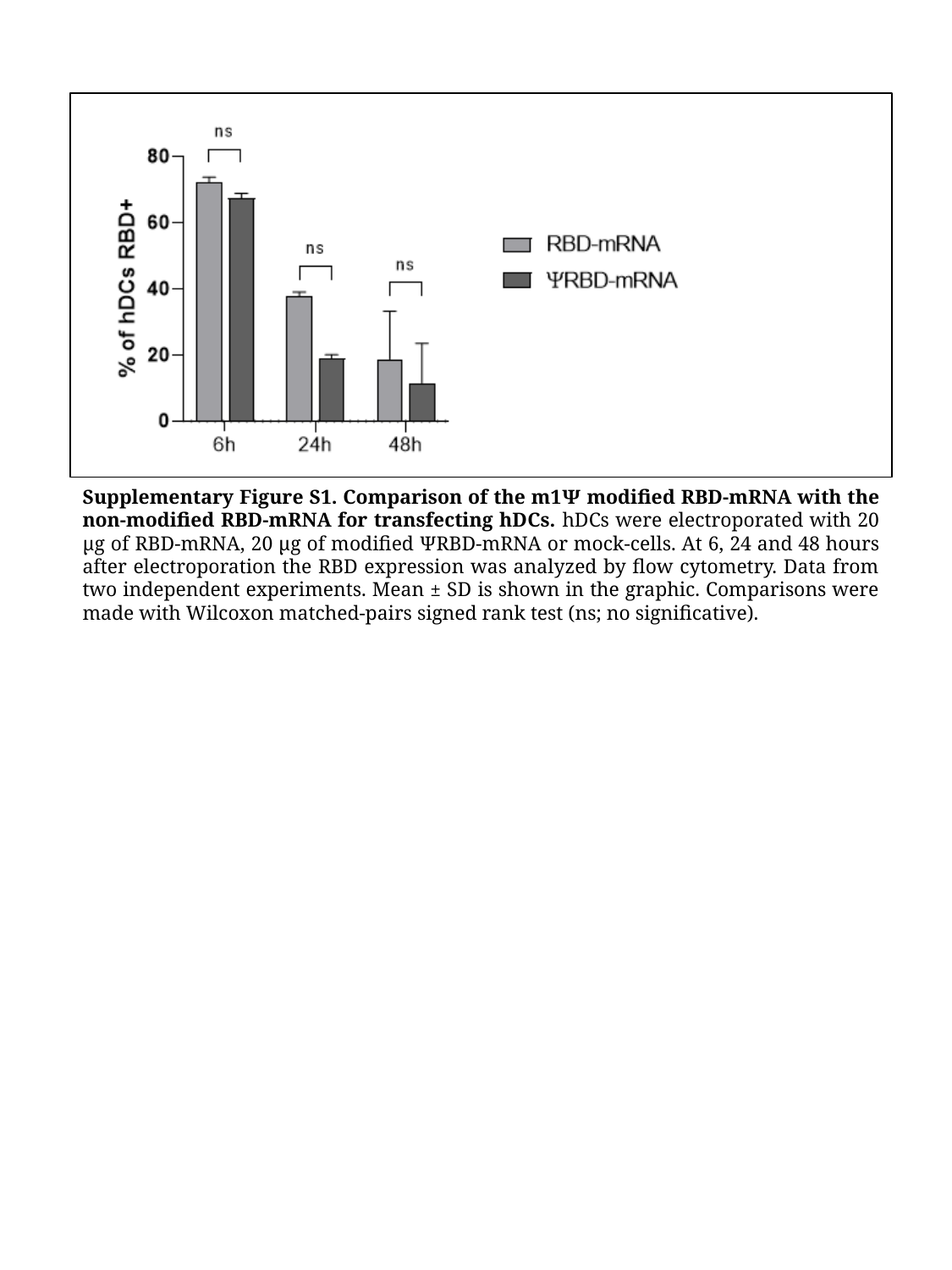

Supplementary Figure S1. Comparison of the m1Ψ modified RBD-mRNA with the non-modified RBD-mRNA for transfecting hDCs. hDCs were electroporated with 20 µg of RBD-mRNA, 20 µg of modified ΨRBD-mRNA or mock-cells. At 6, 24 and 48 hours after electroporation the RBD expression was analyzed by flow cytometry. Data from two independent experiments. Mean ± SD is shown in the graphic. Comparisons were made with Wilcoxon matched-pairs signed rank test (ns; no significative).

## Slide 2
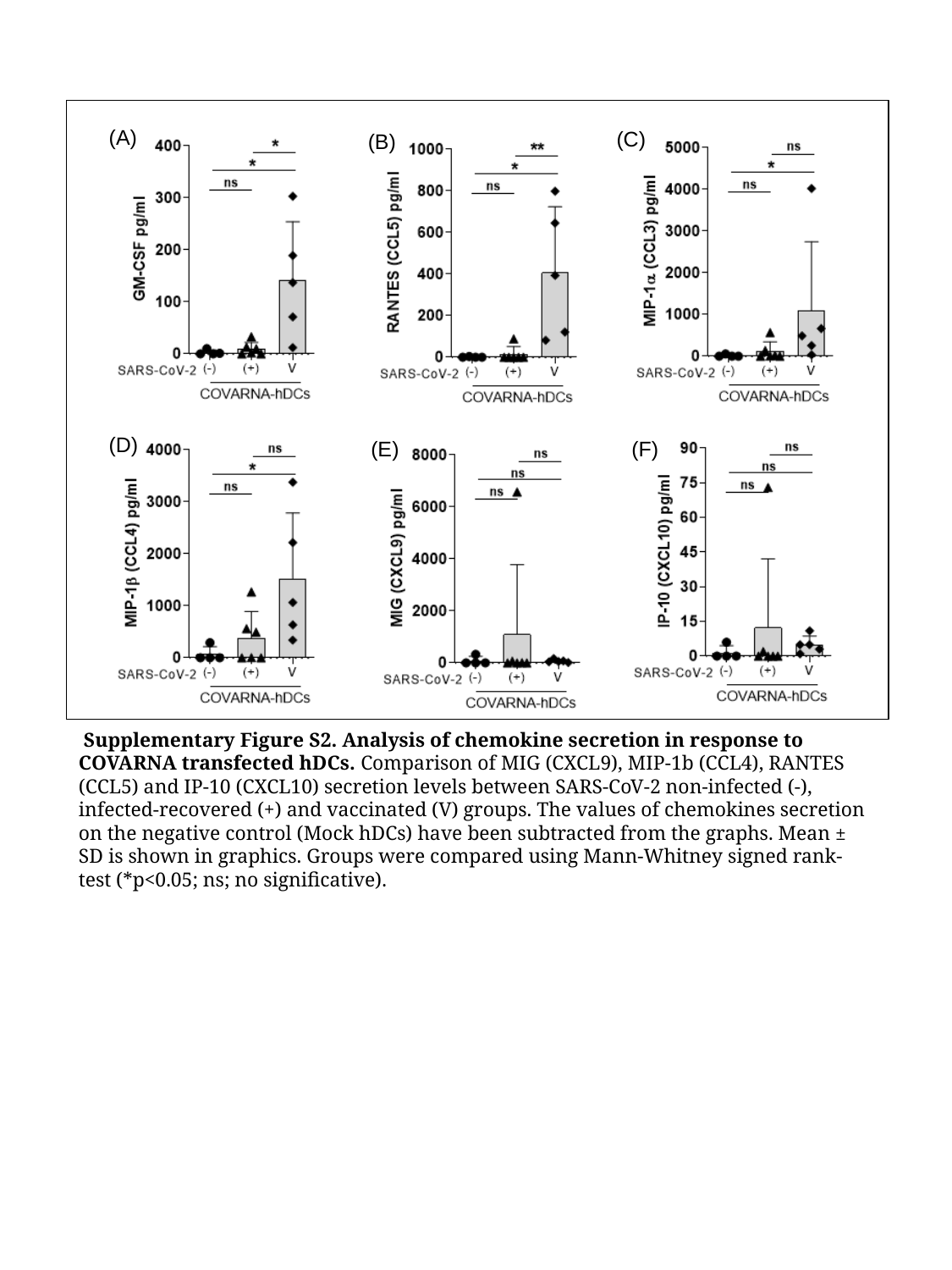

(A)
(C)
(B)
(D)
(E)
(F)
 Supplementary Figure S2. Analysis of chemokine secretion in response to COVARNA transfected hDCs. Comparison of MIG (CXCL9), MIP-1b (CCL4), RANTES (CCL5) and IP-10 (CXCL10) secretion levels between SARS-CoV-2 non-infected (-), infected-recovered (+) and vaccinated (V) groups. The values of chemokines secretion on the negative control (Mock hDCs) have been subtracted from the graphs. Mean ± SD is shown in graphics. Groups were compared using Mann-Whitney signed rank- test (*p<0.05; ns; no significative).

## Slide 3
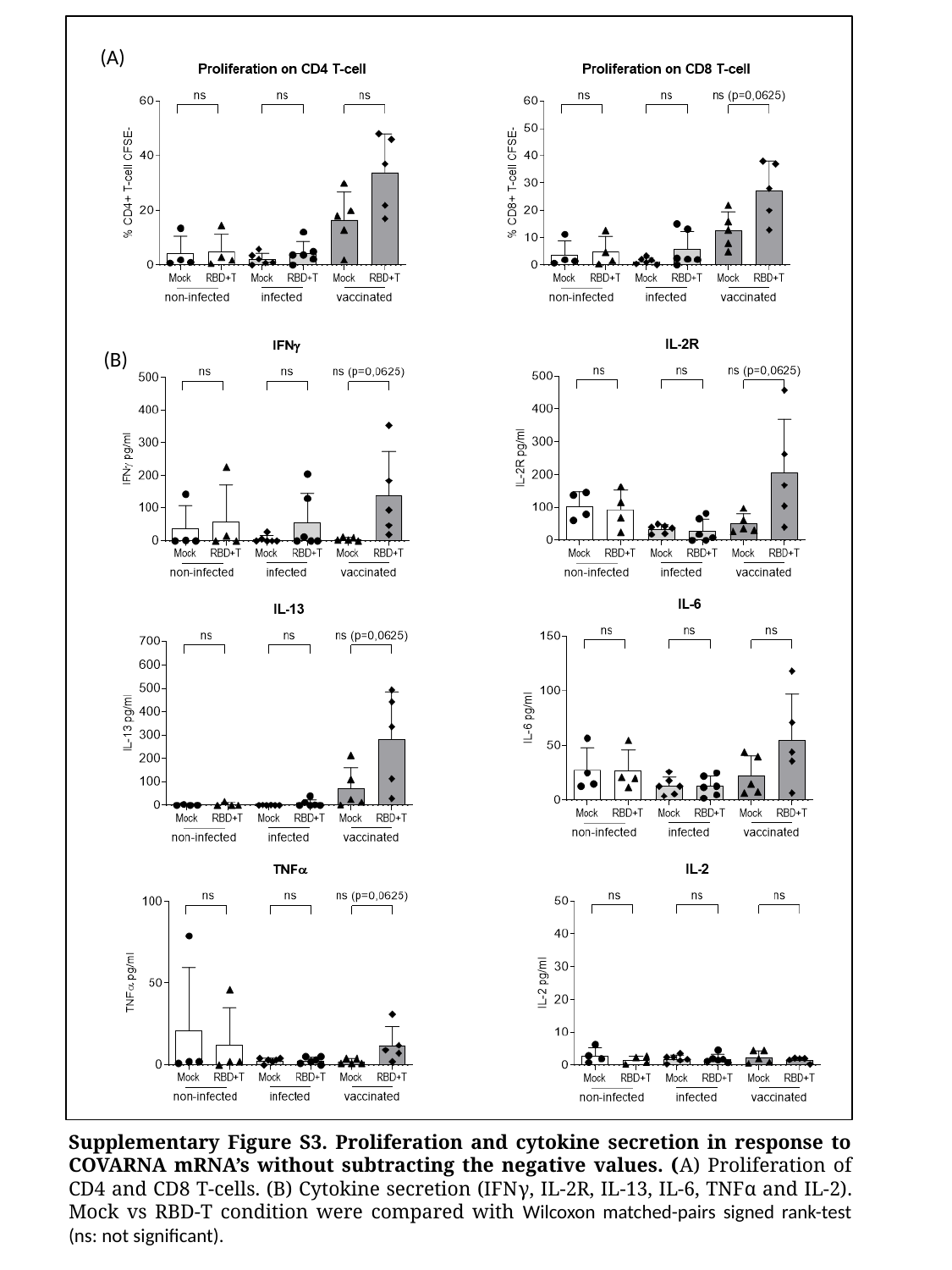

(A)
(B)
Supplementary Figure S3. Proliferation and cytokine secretion in response to COVARNA mRNA’s without subtracting the negative values. (A) Proliferation of CD4 and CD8 T-cells. (B) Cytokine secretion (IFNγ, IL-2R, IL-13, IL-6, TNFα and IL-2). Mock vs RBD-T condition were compared with Wilcoxon matched-pairs signed rank-test (ns: not significant).

## Slide 4
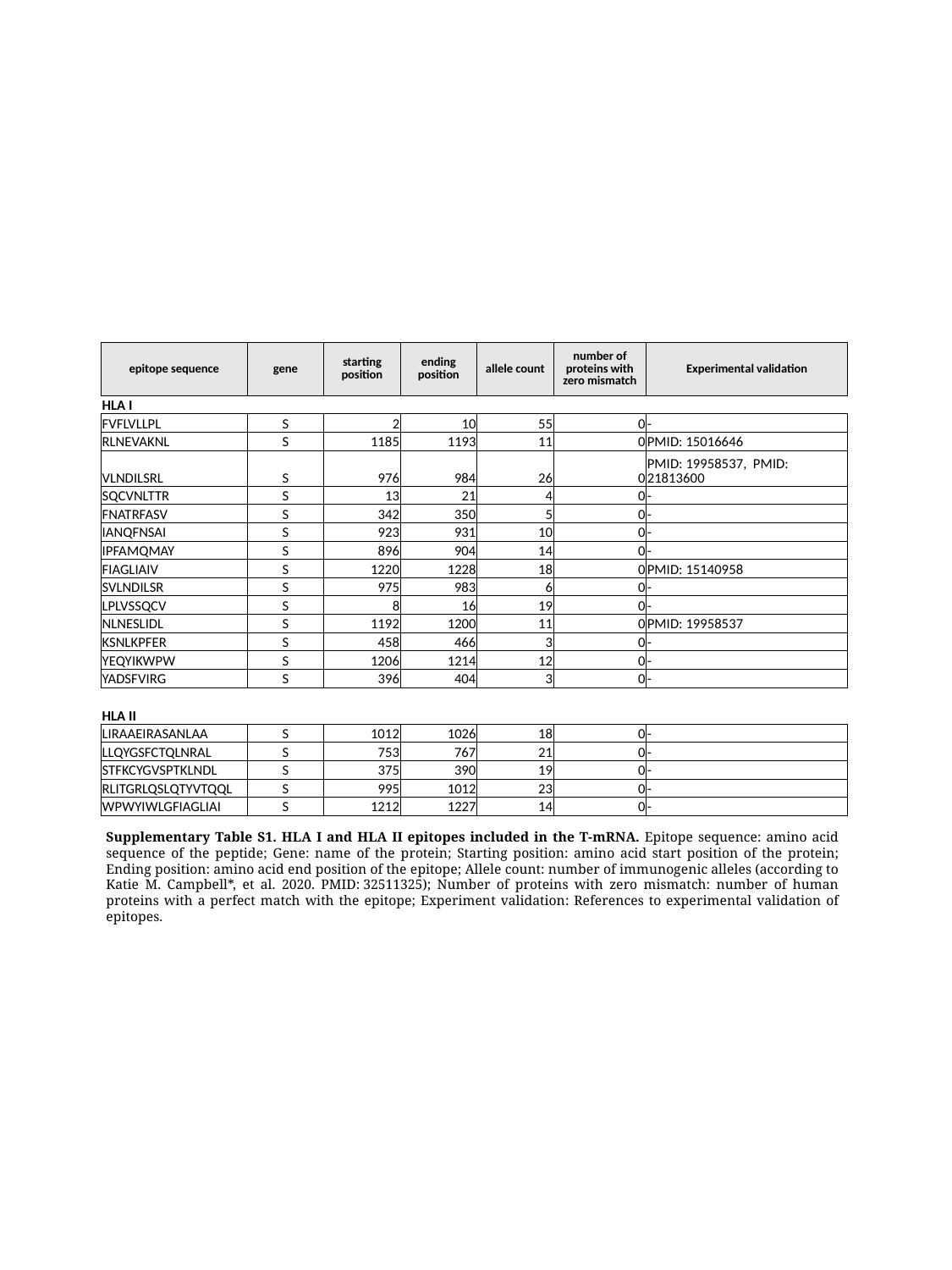

| epitope sequence | gene | starting position | ending position | allele count | number of proteins with zero mismatch | Experimental validation |
| --- | --- | --- | --- | --- | --- | --- |
| HLA I | | | | | | |
| FVFLVLLPL | S | 2 | 10 | 55 | 0 | - |
| RLNEVAKNL | S | 1185 | 1193 | 11 | 0 | PMID: 15016646 |
| VLNDILSRL | S | 976 | 984 | 26 | 0 | PMID: 19958537, PMID: 21813600 |
| SQCVNLTTR | S | 13 | 21 | 4 | 0 | - |
| FNATRFASV | S | 342 | 350 | 5 | 0 | - |
| IANQFNSAI | S | 923 | 931 | 10 | 0 | - |
| IPFAMQMAY | S | 896 | 904 | 14 | 0 | - |
| FIAGLIAIV | S | 1220 | 1228 | 18 | 0 | PMID: 15140958 |
| SVLNDILSR | S | 975 | 983 | 6 | 0 | - |
| LPLVSSQCV | S | 8 | 16 | 19 | 0 | - |
| NLNESLIDL | S | 1192 | 1200 | 11 | 0 | PMID: 19958537 |
| KSNLKPFER | S | 458 | 466 | 3 | 0 | - |
| YEQYIKWPW | S | 1206 | 1214 | 12 | 0 | - |
| YADSFVIRG | S | 396 | 404 | 3 | 0 | - |
| | | | | | | |
| HLA II | | | | | | |
| LIRAAEIRASANLAA | S | 1012 | 1026 | 18 | 0 | - |
| LLQYGSFCTQLNRAL | S | 753 | 767 | 21 | 0 | - |
| STFKCYGVSPTKLNDL | S | 375 | 390 | 19 | 0 | - |
| RLITGRLQSLQTYVTQQL | S | 995 | 1012 | 23 | 0 | - |
| WPWYIWLGFIAGLIAI | S | 1212 | 1227 | 14 | 0 | - |
Supplementary Table S1. HLA I and HLA II epitopes included in the T-mRNA. Epitope sequence: amino acid sequence of the peptide; Gene: name of the protein; Starting position: amino acid start position of the protein; Ending position: amino acid end position of the epitope; Allele count: number of immunogenic alleles (according to Katie M. Campbell*, et al. 2020. PMID: 32511325); Number of proteins with zero mismatch: number of human proteins with a perfect match with the epitope; Experiment validation: References to experimental validation of epitopes.
